# Can Parkinsonism be a solitary manifestation of Wilson’s Disease?

**DOI:** 10.1016/j.prdoa.2025.100402

**Published:** 2025-10-30

**Authors:** Mehri Salari, Fatemeh Hojjatipour, Kamran Rezaei, Masoud Etemadifar

**Affiliations:** a Neurology Department, School of Medicine, Shahid Beheshti University of Medical Sciences, Tehran, Iran; b School of Medicine, Shahid Beheshti University of Medical Sciences, Tehran, Iran; c Neurology Department, School of Medicine, Isfahan University of Medical Sciences, Isfahan, Iran

**Keywords:** Wilson’s Disease, Parkinsonism, Tremor, Idiopathic Parkinson’s Disease

## Abstract

Wilson’s disease (WD) is a secondary parkinsonian condition that results in multi-organ dysfunction. Accurately diagnosing this disorder as soon as possible is crucial since it is a treatable condition. In order to diagnose WD, a doctor must be aware of the disorder’s early signs and request the following diagnostic tests. These include serum ceruloplasmin levels, 24-hour urinary copper excretion, slit-lamp examination for Kayser-Fleischer rings, liver biopsy, and ATP7B genetic testing when needed. It is mentioned that WD can present with a wide range of manifestations; however, according to our observations of thoroughly analyzing cases and papers concerning WD patients, we conclude that parkinsonism cannot be the only early symptom of WD. Most patients who developed parkinsonian symptoms also showed accompanying features such as dysarthria, dystonia, or behavioral changes. This finding would assist doctors in rolling out WD diagnosis and focusing on other potential diseases. By avoiding unnecessary investigations for WD in cases of isolated, typical parkinsonism, physicians can more effectively allocate resources and explore other more probable differential diagnoses. This situation allows for earlier initiation of appropriate therapy tailored to the correct underlying condition, and the overall financial burden on individuals and health systems would be lessened. Such an approach would also help reduce patient anxiety caused by uncertainty.

## Highlights

• Isolated, typical Parkinsonism (like that in iPD) as the sole initial symptom of WD is extremely rare or undocumented.
• Most WD patients with parkinsonian symptoms also have other features such as dystonia, tremor, or psychiatric symptoms.
• Genetic WD testing is not needed in patients with solitary, typical parkinsonism, especially if no red flags are present.
Targeted testing for WD should be based on other features rather than age or parkinsonism alone.

## 1 Introduction

Wilson's disease (WD), an autosomal recessive disorder, occurs due to the inability to metabolize copper effectively, and the extra copper builds up in several organs [1,2]. Consequently, the impairment of an organ exhibits certain symptoms [3]. The diagnosis of this condition must be made as soon as possible, since it is treatable [1,3]. Clinical symptoms, imaging, and biochemical tests help us to diagnose WD [1]. However, the average reported time from symptom onset to diagnosis and treatment initiation is 12 months, though in some cases, the delay may be several decades [2]. Therefore, misdiagnosis and delays in treatment start are common, yet early diagnosis and timely treatment initiation depend on the identification of early clinical characteristics [3].

Non-neurologic manifestations include hepatic disease, renal injury, hemolytic anemia, and Kayser-Fleischer rings. Clinical neurological manifestations include: extrapyramidal manifestations, pyramidal features, cognitive impairments, and psychiatric symptoms [1]. According to the symptoms, patients were categorized as “pure hepatic,“ ”hepato-neurologic,“ ”neurologic,“ ”pure psychiatric,“ ”osseomuscular,“ and ”presymptomatic.“ [3].

Although not all individuals with neurologic WD have cirrhosis, the majority have underlying hepatic disease, and Neurological involvement often appears about10 years after liver involvement. Neurologic WD is often accompanied by psychiatric symptoms [4,5].

Parkinsonism, which presents symptoms such as tremors and slowness, can resemble WD, particularly in younger individuals [6,7]. Medical guidelines, such as those from the American Association for the Study of Liver Diseases (AASLD), suggest that individuals under 40 exhibiting parkinsonian symptoms should be screened for WD using tests like serum ceruloplasmin and urine copper levels [6]. This ensures early detection and is crucial for preventing severe outcomes [1,6]. On the other hand, the occurrence of isolated parkinsonism in WD is uncommon, with fewer than 2 % of presentations matching this characterization [8], and this rarity forms the basis for our question: Does a sole typical manifestation of parkinsonism, without other motor manifestations and non-neurological WD features, necessitate testing for WD?

The present paper aims to review the literature for case descriptions of neurological WD patients and determine whether typical parkinsonism, if present, is a sole manifestation in a WD patient. Additionally, if it were a sole manifestation, we would check the confidentiality of the description as a typical parkinsonism without symptoms potentially compatible with other motor manifestations, challenging the mark of typical parkinsonism for the patient.

## 2 Search strategy

We assess databases such as PubMed, Google Scholar, and Scopus in this study. Wilson's disease, Parkinsonism, or Parkinsonian were the terms we utilized. After examining the abstract and title, we were able to include 142 of the 1061 papers that were originally included. Only 36 papers had sufficient data to be included in our analysis after the entire texts of the included research were evaluated.

**Challenges in identifying isolated typical Parkinsonism in WD patients:** Even though the population studies of WD cases across the literature mostly mention the symptom of diagnosis, most did not differentiate the symptom of onset [9,10]. Research classifying early neurologic symptoms shows that dysarthria is the most prevalent, followed by dystonia, abnormal gait, tremor, parkinsonism, chorea or athetosis, and seizures. Showing that the majority of the WD cases presented with symptoms, rather than being typical of idiopathic Parkinson’s Disease (iPD) presentation [4,11,12]. Interestingly, it is mentioned that neurological manifestations of WD almost always coexist with some behavioral, cognitive, or even psychotic syndromes [13]. However, it is possible for a single manifestation, such as tremor, dysarthria, and dystonia, to be present in isolation at the onset of symptoms, but unilateral rest tremor, chorea, cerebellar findings, and parkinsonism are rarely isolated clinical features of WD [[14], [15], [16]].

The KF rings can be found in almost all WD patients with neurological dysfunction, which can be helpful in the diagnosis of WD in patients with isolated Parkinsonism [5,17]. Furthermore, the diagnosis of neuropsychiatric symptoms in WD needs to be followed by K-F rings, a low ceruloplasmin level in the blood, or an increased 24-hour urine copper level. Moreover, after the lack of a positive result from all of the above-mentioned tests, liver biopsy to measure the amount of copper in the liver and /or genetic testing to determine whether an ATP7 B mutation is present are other indicative tests [18].

Tremor: Tremor is the first clinical manifestation in up to 55 % of WD patients with neurologic symptoms. Tremor can be postural or kinetic. It can also resemble rubral tremor, essential tremor (ET), dystonic tremor, and, in rare cases, Parkinsonian resting tremor. In WD, the most particular and prominent tremor is called wing-beating tremor, which is posture-induced (usually after 30 to 60 s elicited by abduction of the arms, with flexed elbows and palms facing downward, irregular, coarse, of large amplitude, and involves the proximal portions of the upper extremities). In most secondary parkinsonian disorders, including WD, like iPD, rest tremor and rigidity are asymmetrical [13]. Wilson's disease rarely has unilateral rest tremor and parkinsonism as isolated clinical features, but when rest tremor is present, it is typically accompanied by postural and kinetic tremor, which may be more severe than the rest tremor [14]. Subsequently, tremor is almost invariably associated with other neurological signs [19]. A study by Oder and colleagues stated that among 27 neurological WD cases, there was no typical initial symptom resembling PD [20]. There was no initial symptom of bradykinesia or rigidity, and among 10 patients with an initial presentation of tremor, six had posture tremor, three had writing tremor, and the last one had an action tremor [20]. Meanwhile, in another study on 78 WD patients conducted by Pulai et al., a non-differentiated hand tremor was mentioned as the initial presentation of almost one-fifth of the patients, and no bradykinesia or rigidity was present in the list of common initial symptoms [11]. Lack of a precise differentiation of hand tremor in this study and many other population-based WD studies makes it difficult to conclude a relation between typical symptoms of typical Parkinsonism and WD, notably in a setting where other accompanying signs and symptoms are not clearly discussed [11,21].

Although isolated resting tremor is relatively rare as the initial manifestation of WD patients, a case report mentioned that isolated resting tremor of the foot with low frequency was the only presentation of a WD patient; however, serum biomarkers and genetic findings made the diagnosis possible [22]. Furthermore, a 29-year-old lady with upper limb tremor was described in another case report. Over the course of four months, the tremor spread to all four limbs. Later on, she became manic. The presence of KF rings and low serum ceruloplasmin after five years of tremor onset suggested a diagnosis of WD [23]. Also, rest tremor and postural tremor, with ‘wing beating’ tremor, were present in a 33-year-old male with a positive KF ring, changes in MRI, and abnormal serum biomarkers [24]. In another case report, a woman of 38 years old arrived with a resting tremor in her arm, which was followed by stiffness, clumsiness, and dystonia in the same limb. Following the investigation, liver biopsies and increased 24-hour urine copper excretion were found. Additionally, WD was genetically shown by ATP7 B. On the other hand, the 18-Fluorodopa-PET brain scan revealed a classic PD indication. Finally, they came to the conclusion that the patient had both PD and WD at the same time, which supported our hypothesis that WD patients don't present with isolated parkinsonism. Her first manifestations were diagnostic of PD symptoms in her first referral. [17].

**Parkinsonism**: Between 19 and 62 % of WD patients have been reported to have parkinsonism, usually bradykinesia, cogwheel rigidity, or imbalance [14]. In a study by Soltanzadeh and colleagues, although 15 WD cases of parkinsonism were classified as typical, the majority of them (10cases) had drooling and dysarthric phenomena, probably compatible with a dystonic tremor [25]. Meanwhile, a detailed description of each case was not provided in this study, however, a relatively high proportion of cases initially presented with dysarthria (46 %), drooling (22 %), undifferentiated abnormal gait (24 %), and dystonic limbs (24 %), makes it possible that in the cases with parkinsonism, majority (if not all) had a symptom that is not seen in iPD, [25]. According to studies and published cases until 2025, solitary parkinsonism is an uncommon symptom of Wilson's disease; no Wilson patients showed isolated parkinsonism as their initial symptom. Despite the fact that a study found that parkinsonism was the initial presentation in 11 % of patients, or in another study, 25 % of patients, it is neither mentioned to be isolated nor typical of iPD in these patients [16,24].

Due to their precise case description, case reports provide a suitable source for our research. In a case report conducted by Sapuppo et al., two sisters with WD were reported, in whom one was presented with neurological WD [26]. This patient initially presented with the symptoms of dysarthria and possible limb dystonia, with an undifferentiated asymmetrical hand tremor that was prominent during writing [26]. They didn’t clarify the neurological examination of her gait impairment, but they described it as “awkwardness”, which is more compatible with a dystonic or cerebellar gait, rather than a bradykinesia-related walking difficulty. Generally, the presence of a directly mentioned dystonia or dysarthria is a common finding in WD case reports across the literature [[27], [28], [29]]. Additionally, parkinsonism is also mentioned to be the sole neurological manifestation in a WD patient with a long history of psychiatric problems. This is consistent with the idea that isolated parkinsonism cannot be the initial manifestation of WD [30].

## 3 Discussion

Due to WD’s ability to cause a variety of movement problems, it can imitate many disorders, and sometimes it is difficult to diagnose. Furthermore, better diagnostic tools, greater awareness, and a clear differentiation from illnesses that resemble it result in earlier detection, even in the presymptomatic stage [3]. Diagnosis of WD is expensive and challenging since none of the laboratory markers of copper accumulation is 100 % meaningful and specific for WD. [31] One of the tests that can help us diagnose WD is urinary copper excretion. However, this test is also not very trustworthy. This is because the cut-off value considered for WD diagnosis varies in the literature. Urinary copper excretion greater than 100 µg/24 h is accepted as diagnostic for WD, according to the European Association for the Study of the Liver [32,33]. Nonetheless, 40 μg/24 h and greater is also mentioned as another cut-off value [34]. On the other hand, a cut‐off of 0.64 to 1.6 μmol/24 h is used in the Leipzig criteria [35]. Another aspect of WD that makes its diagnosis challenging is the neurological form of this disorder that can resemble PD. This is because WD can induce symptoms that are frequent in PD, such as slowness of movement, hypomimia, shuffling gait, decreased fine finger motions, and foot tapping [36].

The cerebral manifestations in WD patients, such as dyskinesia, dysarthria, tremor, and stiffness, are the result of copper deposition in the substantia nigra or the striatum itself, which is the most likely cause of concordant pre- and postsynaptic dopaminergic impairments in neurologic WD that cause parkinsonism symptoms [37].

Although it is proposed to roll out WD in early onset PD, it is not suggested for Late-onset PD according to MDS criteria [38], and other studies for PD diagnosis [7,38,39]. Nonetheless, it is stated by Bandmann et al. [36] WD diagnosis cannot be limited by age because it can occur in infancy or even in people who are too old. Therefore, we dig deeper to see whether it is necessary to order a WD workup in all EOPD.

Based on our observations and review of the literature on neurological WD, it appears that patients who meet the MDS criteria for PD diagnosis and do not exhibit any red flags for PD (such as dystonia, K-F, or brain imaging that indicates WD) do not require additional testing for WD diagnosis at any age. On the other hand, in contrast to the usual neuropsychiatric PD presentations (depression, anxiety, apathy, hallucinations, and impulse control disorders [40], WD evaluations should be taken into consideration if the patient exhibits atypical neuropsychiatric presentations for PD, such as behavioral and personality changes, anxiety, depression, manic and hypomanic syndrome, cognitive deficits, sleep problems (dyssomnias), and sexual dysfunctions, including excessive sexual drive [41].

In earlier research and papers about the first manifestations of WD, isolated typical symptoms of iPD in WD patients have not been documented. Where parkinsonism was cited as the initial manifestation, it either occurred after atypical mental issues or the symptoms that were not characteristic of iPD. Moreover, according to what we have concluded in WD manifestation, parkinsonism is often accompanied by other symptoms, seen throughout the course of the disease, and is mostly of the akinetic-rigid subtype with a symmetric distribution and bulbar symptoms (drooling, dysphagia, and dysarthria) [15].

The significance of these findings lies in their ability to assist neurologists in making an accurate diagnosis as quickly as feasible. According to previous studies, WD and iPD can be misdiagnosed, hence, more laboratory tests are suggested to be used in cases when a definitive diagnosis is not attainable. This approach could significantly reduce diagnostic uncertainty for both patients and physicians. Moreover, it may lower the overall cost of diagnosis by minimizing the need for extensive laboratory and imaging testing, thereby decreasing the financial burden on patients, healthcare systems, and the broader economy. In this case, doctors will also take into account alternative diagnoses, which can help begin the right treatment for people with movement issues who do not have WD. Further study is needed to support the hypothesis that WD may not initiate with isolated iPD symptoms. This is the first study to bring up such an important matter for future evaluation and studies.

There are some limitations that needs to be addressed in this study. A few of the included publications lacked thorough neurological tests to be suggestive of parkinsonism. Also, it was difficult to distinguish between symptoms at diagnosis and those that occurred initially, which could have resulted in a misclassification. Furthermore, it may be difficult to determine the actual prevalence of atypical or solitary parkinsonism in WD due to underreporting of isolated and unique WD presentations. Moreover, the certainty of conclusions about iPD was limited in a number of instances because tremor and parkinsonism were not easily distinguished from dystonia or other movement disorders.

In conclusion, after evaluating previous studies and observations, we couldn’t find proof of WD that starts with typical isolated parkinsonism, which brings us to the point that WD testing may not be necessary in these cases. However, future assessments are required to replicate our results and confirm this theory.

**Funding**

This research did not receive any specific grant from funding agencies in the public, commercial, or not-for-profit sectors.

**Ethical compliance statement**

• Shahid Beheshti University of Medical Sciences ethics committee approved the study.
• Informed patient consent was not necessary for this work.
• We confirm that we have read the Journal’s position on issues involved in ethical publication and affirm that this work is consistent with those guidelines.

## CRediT authorship contribution statement

**Mehri Salari:** Writing – review & editing, Supervision, Project administration, Methodology, Investigation, Conceptualization. **Fatemeh Hojjatipour:** Writing – review & editing, Writing – original draft, Methodology, Investigation, Data curation, Conceptualization. **Kamran Rezaei:** Writing – original draft, Investigation. **Masoud Etemadifar:** Writing – review & editing, Supervision, Project administration, Investigation, Conceptualization.

## Declaration of competing interest

The authors declare that they have no known competing financial interests or personal relationships that could have appeared to influence the work reported in this paper.
